# Sex differences and estrogen effects in cardiac mitochondria in human aortic stenosis and in the mouse heart

**DOI:** 10.3389/fendo.2023.1181044

**Published:** 2023-10-17

**Authors:** Daniela Fliegner, Alexandra Ellieva, Anja Angelov, Georgi Petrov, Vera Regitz-Zagrosek

**Affiliations:** ^1^ Institute of Gender in Medicine, Charité Universitätsmedizin Berlin, Berlin, Germany; ^2^ Medical Affairs Internal Medicine, Pfizer Pharma GmbH, Berlin, Germany; ^3^ University of Witten- Herdecke, Witten, Germany; ^4^ Clinic for Cardiology, University Hospital Zürich, Zürich, Switzerland

**Keywords:** sex differences, aortic stenosis, cardiac mitochondrial function, estrogen, estrogen receptor modulation

## Abstract

**Introduction:**

Sex differences in the adaptation to pressure overload have been described in humans, as well as animal models, and have been related to sex-specific expression of mitochondrial genes. We therefore tested whether sex differences in cardiac mitochondrial respiration exist in humans with aortic stenosis (AS). We also examined whether these potential differences may be at least partially due to sex hormones by testing if mitochondrial respiration is affected by estrogen (17ß-estradiol (E2)).

**Methods:**

Consecutive patients undergoing transapical aortic valve implantation (TAVI) (women, n = 7; men, n = 10) were included. Cardiac biopsies were obtained during TAVI and used directly for mitochondrial function measurements. Male and female C57BL/6J mice (n = 8/group) underwent sham surgery or gonadectomy (GDX) at the age of 2 months. After 14 days, mice were treated once with intraperitoneally injected vehicle (placebo), 17ß-estradiol (E2), estrogen receptor alpha (ERα) agonist [propyl pyrazole triol (PPT)], or ER beta (ERβ) agonist (BAY-1214257). Thereafter, mitochondrial measurements were performed directly in cardiac skinned fibers from isolated left ventricles and musculus solei.

**Results:**

Mitochondrial State-3 respiration was higher in female than that in male human heart biopsies (15.0 ± 2.30 vs. 10.3 ± 2.05 nmol/mL/min/mg, p< 0.05). In the mouse model, mitochondrial State-3 respiration decreased significantly after GDX in female (27.6 ± 1.55 vs. 21.4 ± 1.71 nmol/mL/min/mg; p< 0.05) and male hearts (30.7 ± 1,48 vs. 23.7 ± 2,23 nmol/mL/min/mg; p< 0.05). In ovariectomized female mice, E2 and ERβ-agonist treatment restored the State-3 respiration to intact placebo level, whereas ERα-agonist treatment did not modulate State-3 respiration. The treatment with E2, ERα-, or ERβ-agonist did not modulate the State-3 respiration in GDX male mice.

**Conclusion:**

We identified sex differences in mitochondrial respiration in the diseased human heart. This is in alignment with known sex differences in the gene expression and proteome level at the functional level. E2 and ERβ affect cardiac mitochondrial function in the mouse model, suggesting that they may also contribute to the sex differences in the human heart. Their roles should be further investigated.

## 1 Introduction

Myocardial hypertrophy (MH) is a leading cause of heart failure (HF) and cardiovascular mortality and morbidity in the Western world. Human aortic stenosis (AS) is characterized by MH, which may progress to HF if not treated surgically in time. Women with isolated AS develop more concentric and less eccentric left ventricular hypertrophy (LVH) and develop less HF than men in similar disease states (1, 2). A faster regression after aortic valve replacement (AVR) was observed in women compared with men (3, 4), suggesting sex differences in the adaptation to pressure overload and LVH regression (2, 3). Sex differences in myocardial gene expression and protein composition in human AS are particularly related to mitochondrial gene expression and protein composition (5). Sex differences are mostly caused by sex hormones or unequal gene expression from sex chromosomes or autosomes. Estrogen 17ß-estradiol (E2) and ERs have been described as prime candidates to mediate sex differences in myocardial remodeling (6). Both ERs, ER alpha (ERα) and ER beta (ERβ), are expressed in the human heart, and their upregulation in hearts with AS suggests a functional role (6).

Previous experiments in pressure overload mouse models show major sex differences in cardiac morphology and function (7, 8). Gene expression studies suggest that the observed sex differences are related to sex differences in mitochondrial metabolism and myocardial remodeling/collagen turnover (9, 10). However, only a limited number of studies investigated the effects of sex hormones on mitochondrial function. Either sex differences have been described in the heart or the effects of estrogen and progesterone on mitochondrial function and the generation of reactive oxygen species (ROS) have been described, but mostly in the brain (11) and not in the heart (12–14). Consequently, there is still limited knowledge about the influence of sex and sex hormones on mitochondrial respiration in the cardiovascular system (15). The sex differences in the rodent hearts are similar to changes observed in human hearts, in exhibiting more concentric hypertrophy, less dilatation, and less downregulation of genes involved in energy metabolism in female hearts compared with males as reviewed recently (5, 16). Therefore, the mouse model is suitable to study the mechanistic aspects of sex differences in human mitochondrial function in the heart.

**Figure 1:**
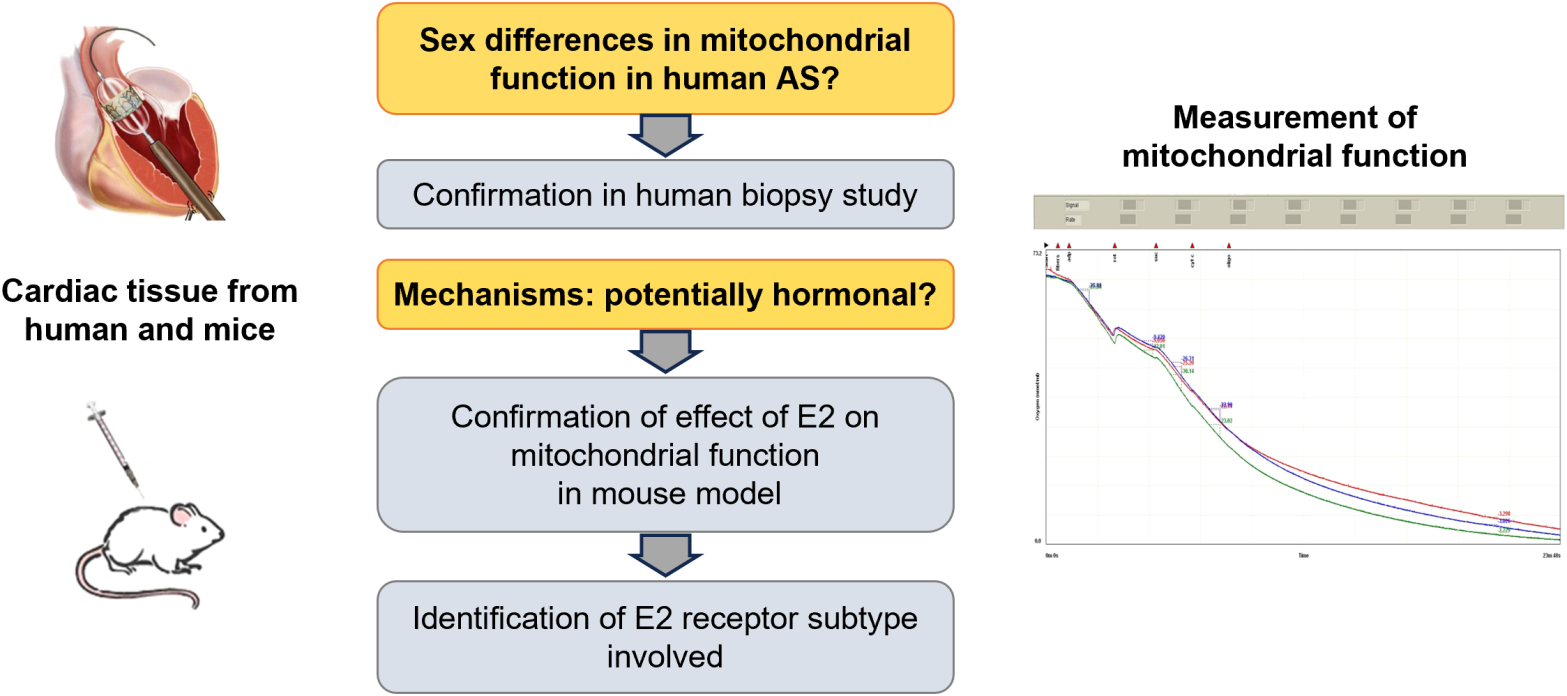
Schematical hypothesis. Main research questions are labeled in orange, main results in grey. Figures at the left depict source of material, figure at the right depicts an exemplary mitochondrial measurement using an oxygraph system. Oxygen consumption was measured in triple.

We hypothesized that female and male patients with AS differ significantly in mitochondrial function and tested this in surgical material from patients undergoing transapical aortic valve implantation (TAVI). To clarify whether these sex differences may be due to the effect of sex hormones, we analyzed whether endogenous or exogenous estrogens have an effect on mitochondrial function in a mouse model (**Figure 1**).

## 2 Materials and methods

### 2.1 Human study population

Patients with AS and who were eligible for TAVI and provided written informed consent were included in a consecutive manner into the study. This study was approved by the Charité University Hospital Ethics Committee and complies with the principles outlined in the Declaration of Helsinki.

The severity of AS was assessed using Doppler echocardiography, in accordance with the guidelines and as described in detail by Baumgartner et al. (17, 18). Cardiac function was assessed 1 week before TAVI and from 3 to 7 days postoperatively. A severe AS was observed in all patients and in both sexes. No significant sex difference was observed in the severity of AS within the sample cohort. Patients’ basic clinical characteristics are presented in **Table 1**. Myocardial biopsies from the left ventricle (apex) were obtained during cardiac surgery. This myocardial biopsy (average size 5 mm) was collected and used directly for the immediately following mitochondrial measurements. In total, myocardial biopsies from 17 patients (7 women/10 men) with AS undergoing TAVI were obtained and included in the present study.

**Table 1:** Basic and clinical characteristics of the human study population (mean ± SEM).

	women	men	p-value
	(n = 7)	(n = 10)	
Age (years)	80.1 ± 7.9	76.7 ± 6.1	0.1632
Body surface area (BSA, m^2^)	1.63 ± 0.06	1.97 ± 0.11	< 0.0001
Body mass index (BMI, kg/m^2^)	23.97 ± 4.9	27.68 ± 0.9	0.0308
CAD 1	28,6%	30,0%	
CAD 2	42,9%	10,0%	
CAD 3	28,6%	50,0%	
AS	II, III, AVR	III, AVR	
AI	I, I-II, II	I, II	
NYHA	II - III	II - III	
aHyp	14,3%	60,0%	
pHyp	42,9%	20,0%	
COPD	28,6%	20,0%	
AF	28,6%	50,0%	
CKD	28,6%	40,0%	
PAD	14,3%	20,0%	
Diabetes mellitus type 2	14,3%	60,0%	

CAD, coronary artery disease; AS, aortic stenosis; AI, aortic insufficiency; NYHA, New York Heart Association classification; aHYP, arterial hypertension; pHYP, pulmonary hypertension; COPD, chronic obstructive pulmonary disease; AF, atrial fibrillation; CKD, chronic kidney disease; PAD, peripheral arterial disease.

Selection criteria for the human biopsies: The inclusion criteria were informed consent and availability of basic and clinical characteristics. Exclusion criteria were emergency surgery, second AVR/TAVI, transfemoral AVR, significant coronary artery obstruction (previous or simultaneous coronary artery bypass graft or percutaneous coronary intervention), valvular heart disease (significant, i.e., stage II concomitant valve disease), significant congenital heart disease, uncontrolled hypertension (blood pressure: > 160/> 100 mm Hg), or other noteworthy comorbidities (preexisting cardiomyopathy or myocarditis, pacemaker, endocarditis, or malignancies) or language problems that might affect the clinical outcome or limit informed consent as described in earlier studies (3).

### 2.2 Mouse model

All experiments were carried out in accordance with the EU Directive 2010/63/EU for animal experiments and approved by the *Landesamt für Gesundheit und Soziales*, Berlin, Germany (G0027 - 11) and followed the “Principles of Laboratory Animal Care” (NIH publication no. 86 - 23, revised 1985) as well as the current version of the German Law on the Protection of Animals.

Male and female C57BL/6J mice were used for the animal experiments. They were kept in temperature-controlled rooms with a 12-h light/dark cycle and provided *ad libitum* access to water and phytoestrogen-free food (Sniff, Soest, Germany). For the *in vivo* series, male and female mice were gonadectomized at the age of 8 weeks. Briefly, all animals were anesthetized by inhalation with 1.5% isoflurane and gonadectomy (GDX) was performed as described in detail elsewhere (19, 20). For postoperative analgesia, 5 mg/kg Rimadyl (carprofen) s.c. was injected and the animals were placed under red light until full awakening.

**Figure 2:**
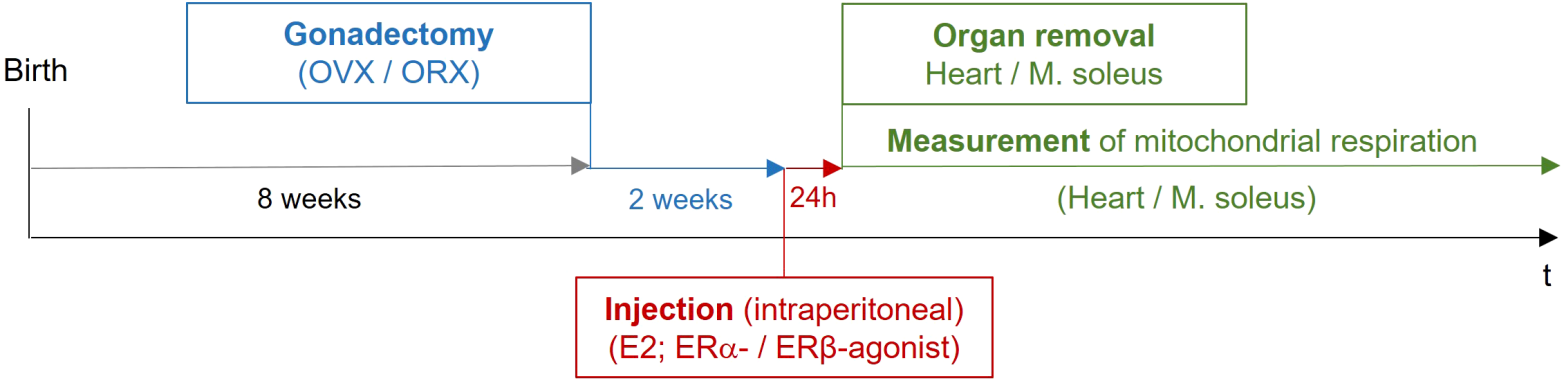
Study design of *in vivo* mouse experiments. Female and male mice at the age of 8 weeks were gonadectomized. Fourteen days after the surgical procedure, the animals were treated once intraperitoneally with vehicle (placebo), estrogen (E2), or with a specific ERα or ERβ agonist. After 24 h, the hearts and the musculus soleus were removed for the measurement of mitochondrial respiration. OVX, ovariectomy; ORX, orchidectomy; E2, 17β-estradiol; ER, estrogen receptor; IP, intraperitoneal.

Fourteen days after the surgical procedure, the animals were treated once intraperitoneally with vehicle (placebo), estrogen 17ß-estradiol (E2), or with a specific ERα or ERβ agonist. After 24 h, the hearts and the musculus soleus were removed for the measurement of mitochondrial respiration (**Table 2**; **Figure 2**).

**Table 2:** Overview animal groups.

Group/Treatment		Female	Male
1	Intact placebo	8	8
2	GDX/Placebo	8	8
3	GDX/E2	8	8
4	GDX/ERα agonist	8	8
5	GDX/ERβ agonist	8	8

GDX, gonadectomy (OVX/ORX); OVX, ovariectomy; ORX, orchidectomy; E2, 17β-estradiol; ER, estrogen receptor.

**Figure 3:**
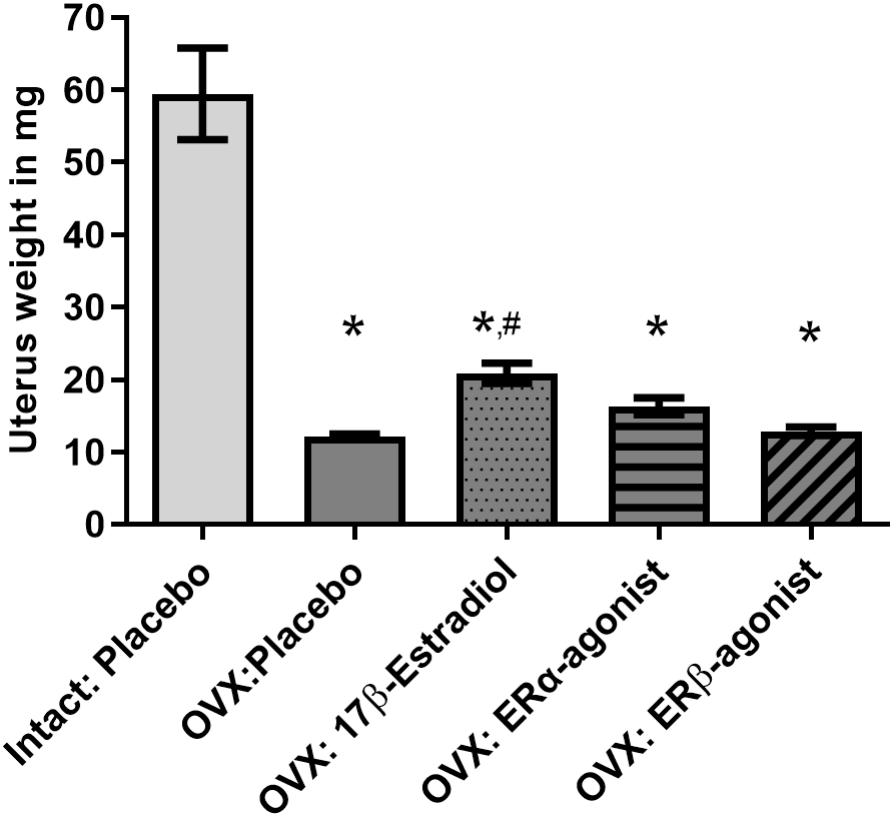
Uterine weights of female mice. Uterine weights are depicted in mg. OVX, ovariectomy; ORX, orchidectomy; E2, 17β- estradiol; ERβ agonist, estrogen receptor beta agonist; ERα agonist, estrogen receptor alpha agonist (PPT, propyl pyrazole triol). One-way ANOVA and Šídák’s multiple comparisons test; *p< 0.05 significant to intact placebo group (female), #p< 0.05 significant to OVX placebo group, n = 8/group. Data present mean ± SEM.

For this purpose, female and male mice were gonadectomized at the age of 8 weeks to remove endogenous sources of ovarian and testicular hormones. The animals were granted a recovery period of 2 weeks postsurgery, facilitating adaptation to the altered hormonal status (clearance of residual hormones and equilibration to the state of hormonal deprivation). The uterine weights were assessed to check the responsiveness to ovariectomy (OVX) (**Figure 3**). The uterine weights from all OVX groups were significantly lower compared to that from the intact placebo-treated group.

The measurements of mitochondrial function in cardiac fibers from both male and female mice were performed in parallel. After sacrificing the mice, the hearts were isolated and cardiac skinned fibers were prepared immediately and mitochondrial function was measured.

#### 2.2.1 Estrogen and ER agonistic stimulation

Two weeks after the GDX, the animals were treated once intraperitoneally with E2 (17β-estradiol, Sigma Aldrich; 2,6 µg per 25 -g mouse), or with a specific ERα agonist [propyl pyrazole triol (PPT), Sigma Aldrich; 5,2 µg per 25 -g mouse], or with a specific ERβ agonist (BAY-121-4257, 2,6 µg per 25 -g mouse). These concentrations were chosen to achieve the E2 level within a physiological range, as we had done previously (21, 22).

At the end of the experiment, all mice were sacrificed. Hearts and musculus solei were isolated for mitochondria respiration measurements, and the uteri have been removed to assess the response to OVX.

### 2.3 Measurements of mitochondrial function

#### 2.3.1 Human cardiac sample preparation

In total, we utilized 17 human cardiac biopsies, 7 female and 10 male apical left ventricular samples. The human heart samples were transferred to ice-cold oxygenized Krebs–Henseleit buffer immediately after collection and rinsed carefully. Subsequently, biopsies were prepared directly for mitochondrial respiratory measurement.

Following the measurement of basal respiration, maximal adenosine diphosphate (ADP)-stimulated respiration is determined by exposing fibers to ADP (State-3) and various substrates such as glutamate (Glu) and malate (Mal). The integrity of the outer mitochondrial membrane was assessed by adding exogenous cytochrome C (CytC) to ADP-stimulated mitochondria. Uncoupled respiration (State-4) was evaluated after the addition of sodium azide to inhibit nonreversible CytC oxidase or after oligomycin administration. The coupling of phosphorylation to oxidation is determined by the ratio of State-3 to State-4 respiration [respiratory control index (RCI)]. Respiration rates are expressed as nanomoles of O2 per minute per milligram dry weight of fibers.

#### 2.3.2 Mouse heart sample preparation

Mice were sacrificed, hearts were removed quickly and placed into oxygenated Krebs –Henseleit buffer and rinsed there carefully. All fat and connective tissue were then removed, and the whole heart weight was determined. Atria and the right ventricle were removed, and only the left ventricle and the septum were used for the next preparation steps. Additionally, musculus soleus samples as controls were prepared for the mitochondrial measurements.

#### 2.3.3 Mitochondrial measurements in cardiac skinned fibers

The mitochondrial measurements of cardiac skinned fiber preparations were performed as described in detail in Kuznetsov et al. (23). Briefly, the mitochondrial functional measurements of skinned cardiac fibers (human or mouse origin) were measured by using oxygraph chambers and Clarke electrodes from Hansatech Instruments. After the first preparation steps described above, cardiac samples were transferred into a Petri dish with an ice-cold buffer solution and the whole “skinning” procedure was performed on ice. The separation of a cardiac bundle into individual fibers that were still connected with each other is a very critical step. The entire procedure should be carried out with great care, yet swiftly enough to maintain the connectivity and viability of the cardiac fibers while preventing any ischemic damage. These fibers were then permeabilized under stirring for 30 min in saponin buffer and then washed twice for 10 min. From the permeabilized fibers, pieces measuring 2 × 2 mm (approximately 3–5 mg wet weight) were isolated and immersed in 1 mL of potassium chloride buffer (KCl 120 mM, KH_2_PO_4_ 5 mM, EGTA 1 mM, HEPES 3 mM, and 1 mg/mL BSA supplemented with 5 mM Glu and 2 mM Mal as substrates) within the oxygraph chambers. The chambers were closed airtight, and the basal respiration of the fibers was determined. Thereafter, the oxygen consumption was recorded and presented as a graph during adding the following substrates and metabolites in an interval of approximately 2 min: 2 mM ADP, 4 μM rotenone, 10 mM succinate, 8 μM CytC, 4 μM antimycin A, 0.5 mM tetramethyl-p-phenylenediamine (TMPD), and 7.5 mM sodium azide. At the end, the RCI, the ratio of maximum respiration (State-3) and respiration in the absence of ADP (State-4), was determined by the software program (12). The oxygen consumption rates from cardiac skinned fibers were measured at a temperature of 25°C. All human samples were measured in duplicate, and the samples from mice were measured in triple each.

### 2.4 Statistics

All data are presented as means ± SEM and were evaluated for normal distribution. All mouse data were tested by one-way ANOVA and Šídák’s multiple comparisons test, and the human data were tested with one -sample t-test and Wilcoxon test by using GraphPad Prism 9.5.1. For all statistical analyses, p -values ≤ 0.05 were considered statistically significant.

## 3 Results

### 3.1 Sex differences in the clinical manifestation in patients with AS

This study included 17 human cardiac tissue samples. The women and men showed similar characteristics and presence of comorbidities. Women and men were of comparable age; the mean age was 78 years. The patients had equal symptoms and New York Heart Association functional class at referral for TAVI and comparable cardiovascular risk profile as reflected by a similar prevalence of hypertension, chronic obstructive pulmonary disease (COPD), and coronary artery disease (CAD) from classes I to III. Body surface area (BSA) and body mass index (BMI) were significantly lower in women than those in men (**Table 1**). Atrial fibrillation and diabetes mellitus type 2 were more prevalent in men (**Table 1**).

LV mass before TAVI was significantly higher in men, but sex differences were diminished after adjustment for BSA. Relative wall thickness (RWT) was significantly higher in women before surgery (**Table 3**).

**Table 3:** Echocardiographic parameter and left ventricular dimension and function determined and calculated from echocardiographic measurements before and after TAVI.

	Women	Men	p-value	Women	Men	p-value
	before TAVI			after TAVI		
LVM (g)	194.0 ± 22.2	256.3 ± 17.1	0.04	173.0 ± 21.4	240.8 ± 38.2	0.12
LVMI (g/m^2^) (LVM/BSA)	119.5 ± 14.7	128.3 ± 8.1	n.s	106.4 ± 14.2	118.6 ± 16.3	n.s
RWT	0.53 ± 0.06	0.40 ± 0.03	0.05	0.52 ± 0.05	0.45 ± 0.06	n.s
LVID_(d)_ (mm)	44.7 ± 2.45	56.1 ± 3.57	0.03	42.5 ± 3.54	51.0 ± 2.78	0.09 (n.s)
LVID_(d)_/BSA (mm/m^2^)	27.4 ± 1.78	28.4 ± 1.57	n.s	26.2 ± 2.48	25.5 ± 1.30	n.s
PWT_(d)_ (mm)	11.2 ± 0.88	10.4 ± 0.76	n.s.	11.0 ± 0.43	11.8 ± 1.10	n.s
LV-IVS_(d)_ (mm)	13.2 ± 0.74	12.0 ± 0.67	n.s.	12.0 ± 0.78	10.5 ± 0.89	n.s
LV-EDD (mm)	44.9 ± 7.50	53.8 ± 8.30	0.04	44.0 ± 7.30	51.5 ± 9.30	n.s
LVEF (%)	62.5 ± 6.19	42.8 ± 6.76	0.06 (n.s.)	63.0 ± 7.78	48.1 ± 7.89	n.s

LV, left ventricle/ventricular; LVM, LV mass; LVMI, LV mass index; RWT, relative wall thickness; LVID_(d)_, LV inner diameter diastole; BSA, body surface area; PWT_(d)_, posterior wall thickness at end diastole; IVS_(d)_, interventricular septum thickness at end diastole; LVEDD, LV end-diastolic diameter; LVEF, LV ejection fraction; TAVI, transcatheter aortic valve implantation. Data present mean ± SD. n.s., not significant.

### 3.2 Sex differences in postoperative recovery and remodeling

After TAVI, the response and recovery seem to be different between the sexes. Based on the left ventricular hypertrophy (LVH) index, LVH was classified as adaptive (mildly to moderately abnormal) or maladaptive (severely abnormal). We observed within this relatively small human study population that women (n = 7) exhibited a more adaptive LVH, while men (n = 10) exhibited a more maladaptive LVH (women: 119,5 g/m^2^ vs. men: 128,20 g/m^2^) preoperatively. This was reflected by slightly greater preoperative mean left ventricular mass (LVM) indexes in men than in women (**Table 3**).

### 3.3 Sex differences in mitochondrial function in cardiac tissues of patients with AS

**Figure 4:**
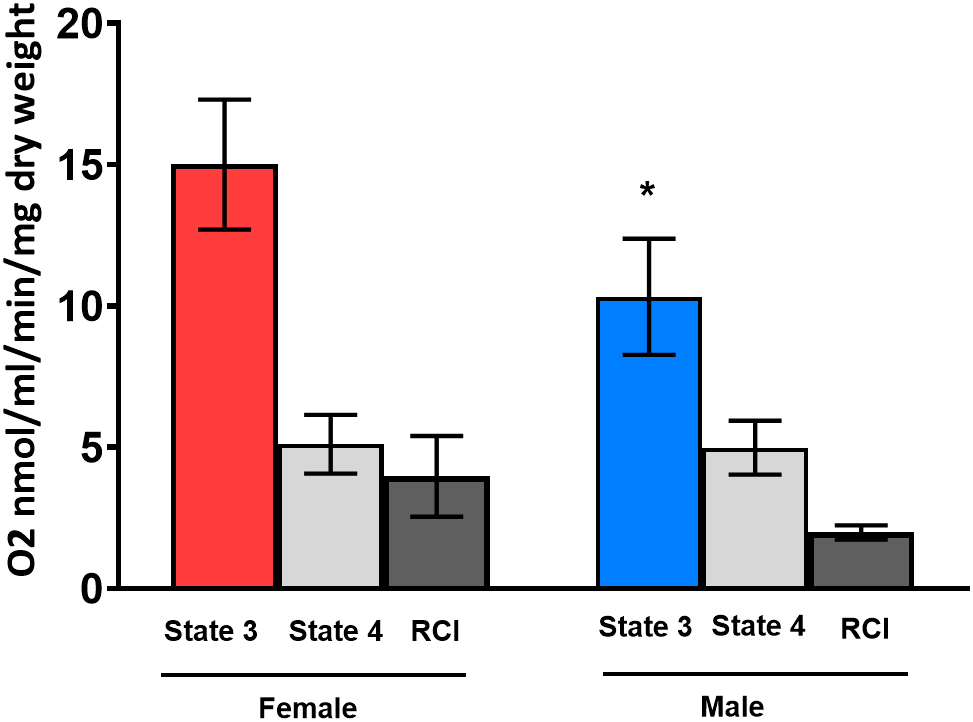
State-3 respiration in cardiac skinned fibers in female (red column) and male (blue column) human hearts. Substrates glutamate and malate (Glu/Mal) were used for the mitochondrial measurements. Oxygen consumption of State-3 respiration is depicted in nmol/mL/min/mg dry weight. dw, dry weight; RCI, respiratory control index. One -sample t-test and Wilcoxon test. *p< 0.05 significant to intact placebo group (female), n = 8/group. Data present mean ± SEM.

We assessed the mitochondrial respiration in human cardiac fibers from patients with AS from both sexes. We observed a significant sex difference in the State-3 respiration. Cardiac respiration from female hearts (15.0 ± 2.30 nmol/mL/min/mg) was significantly higher than male cardiac respiration (10.3 ± 2.05 nmol/mL/min/mg) (**Figure 4**). The State-4 respiration was similar in both sexes (5.1 vs. 4.9 nmol/mL/min/mg) as well as the calculated RCI (3.97 vs. 1.99 nmol/mL/min/mg).

### 3.4 Effect of gonadectomy and E2 and ER stimulation on mitochondrial function

**Figure 5:**
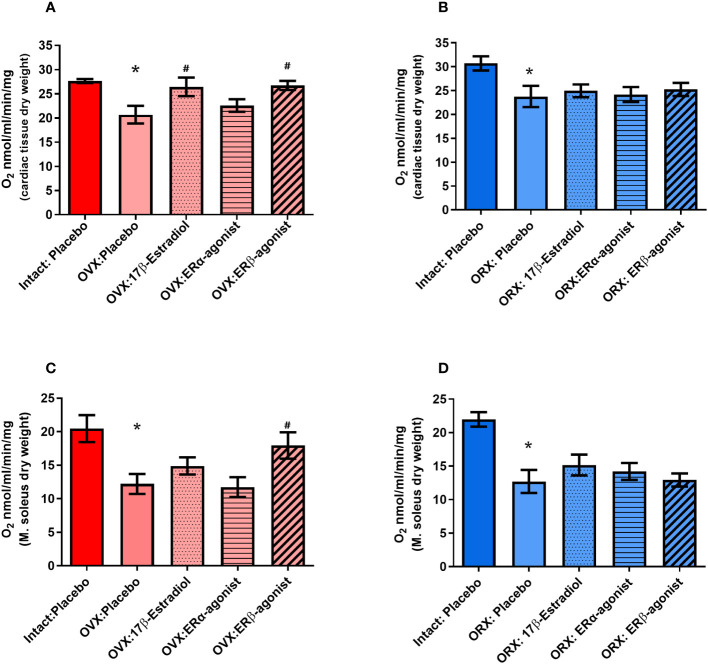
State-3 respiration in cardiac skinned fibers in female **(A)** and male **(B)** mice and skeletal muscle skinned fibers in female **(C)** and male **(D)** mice. Substrates glutamate and malate (Glu/Mal) were used for the mitochondrial measurements. Oxygen consumption of State-3 respiration is depicted in nmol/mL/min/mg dry weight. dw, dry weight; OVX, ovariectomy; ORX, orchidectomy; E2, 17 β -estradiol; ERβ, estrogen receptor beta; ERα agonist, estrogen receptor beta (PPT, propyl pyrazole triol). ANOVA and Šídák’s multiple comparisons test; *p ≤ 0.05 significant to Intact placebo group (female) and Intact placebo group (males), #p< 0.05 significant to OVX placebo group females and ORX placebo group (males), n = 8/group. Data present mean ± SEM.

For this purpose, female and male mice were gonadectomized at the age of 8 weeks to eliminate endogenous sources of ovarian and testicular hormones. Two weeks later, mice were treated with E2 and ER agonists (**Figure 2**; **Table 2**). Cardiac mitochondrial State-3 respiration did not differ between intact female and male hearts (27.6 ± 1.55 vs. 30.7 ± 1,48 nmol/mL/min/mg). However, the State-3 respiration in skinned fibers of cardiac tissue was significantly reduced in all gonadectomized placebo-treated mice compared with their respective intact group (21.4 ± 1.71 vs. 23.8 ± 2.23 nmol/mL/min/mg) (**Figures 5A, B**; **Table 4**). In OVX female mice, E2 and ERβ agonist treatment restored the State-3 respiration toward intact female mice (p ≤ 0.05) (**Figure 5A**; **Table 4**). No significant treatment effect was observed in male mice (**Figure 5B**; **Table 4**). ERα agonist treatment did not modulate mitochondrial respiration in female OVX or male orchidectomy (ORX) mice.

**Table 4:** Detailed State-3 respiration rates from cardiac and skeletal muscle fibers in female and male mice.

	Intact	GDX			
	Placebo	Placebo	E2	ERα-agonist	ERβ-agonist
female heart					
Mean	27,63	21,43*	27,67^#^	22,42	26,82^#^
SD	4,39	4,547	5,11	3,48	2,28
SEM	1,55	1,719	1,93	1,23	0,93
male heart					
Mean	30,7	23,76*	24,95	24,18	25,25
SD	3,92	5,91	3,75	4,121	3,95
SEM	1,48	2,23	1,42	1,557	1,49
female musculus soleus					
Mean	20,46	12,21*	14,89	11,74	17,94
SD	4,91	3,96	3,14	4,29	4,82
SEM	2,01	1,62	1,57	1,92	2,16
male musculus soleus					
Mean	21,97	12,57*	15,16	14,21	12,93
SD	2,65	2,04	4,39	3,30	2,78
SEM	1,08	0,83	1,55	1,25	0,98

GDX, gonadectomy (OVX/ORX); OVX, ovariectomy; ORX, orchidectomy; E2, 17β-estradiol; ER, estrogen receptor. ANOVA and Šídák’s multiple comparisons test. *p ≤ 0.05 significant to intact placebo group (female) and intact placebo group (males), #p< 0.05 significant to OVX placebo group females and ORX placebo group (males), n = 8/group.

The mitochondrial State-3 respiration was also measured in skeletal muscle skinned fibers (musculus soleus). The State-3 respiration did not differ between intact female and male skeletal muscle (20.5 ± 2.01 vs. 21.9 ± 1.08 nmol/mL/min/mg, **Figures 5C, D**; **Table 4**). The State-3 respiration of skeletal muscle skinned fibers was significantly reduced in all GDX placebo-treated mice compared with their respective intact group (12.5 ± 1.62 vs. 12.5 ± 0.84 nmol/mL/min/mg, **Figures 5C, D**; **Table 4**). While no significant treatment effects with E2 and ERα agonist were observed in all ORX males (**Figure 5D**; **Table 4**), only the ERβ agonist treatment increased the State-3 respiration to the placebo/intact level in OVX females (**Figure 5C**; **Table 4**).

In the heart and in the skeletal muscle in both sexes, the mitochondrial State-4 respiration or the calculated RCI showed no difference in the intact placebo -treated mice. The surgical intervention of OVX/ORX and the treatment with E2 or ER agonistic modulation showed no effect on these two parameters (data not shown).

## 4 Discussion

The present study is the first to demonstrate that in patients with AS, the mitochondrial respiration differs significantly between the sexes in the human heart. Specifically, the State-3 respiration was significantly higher in female human cardiac tissue samples compared with that in male samples. Furthermore, an additional significant finding of this study is that sex hormones impact cardiac mitochondrial function, as demonstrated in intact mice. Moreover, the depletion of endogenous sex hormones led to a reduction in mitochondrial respiration in both sexes, which could be restored through treatment with exogenous estrogen and ERβ agonists in female mice.

### 4.1 Sex differences in cardiac metabolism

We found a higher State-3 mitochondrial respiration in female human cardiac skinned fibers compared with their male counterparts. Sex differences in cardiac metabolism specifically in mitochondrial function and the underlying mechanisms have been investigated only rarely in the human heart of both sexes. They may however be related to previously described sex-dependent effects that concern the lower ROS production reported in human and murine female cardiac mitochondria (13), better antioxidant protection (24) and better calcium handling (25) compared with male cardiac mitochondria. These mechanisms contribute to the observed female resilience of HF or slower progression toward HF (14).

Our results showed that the lack of endogenous steroid hormones leads to decreased cardiac and skeletal muscle mitochondrial respiration with substrates Glu/Mal in both sexes after the GDX. Treatment with E2 increased the respiration with substrates Glu/Mal, and the treatment with the ERβ agonist restored the mitochondrial respiration only in female hearts. It became clear that treatment with an ERβ agonist has the same effect as E2 treatment in the heart, as well as in the skeletal muscle. A significantly increased State-3 respiration rate was measured in female OVX mice treated with ERβ agonist and in mice treated with E2 in comparison with their respective control group (OVX/placebo).

Interestingly, reports indicate marked sex differences among healthy individuals regarding mitochondrial function in the heart. In fact, female rat heart mitochondria produce less ROS and have a greater antioxidant capacity than those from males, resulting in a better protection of heart function in females (13).

The observed sex differences in mitochondrial function are fully in agreement with sex differences in cardiac energy metabolism that have been described earlier by us and others (5, 26). Remarkably, a recent unbiased proteomic profiling analysis compared left ventricular myocardial biopsies from patients with different heart valve diseases: AS and mitral valve regurgitation (MR) and controls. The authors found that proteins of important metabolic pathways such as in the tricarboxylic acid cycle are downregulated in AS and MR, while other main transporters for long -chain fatty acids or glucose were upregulated in AS and MR (5). More important sex -stratified analysis among the AS patients revealed that proteins related to mitochondrial function showed a sex-specific pattern: a strong reduction in proteostasis-related proteins and less decrease in proteins involved in energy metabolism in female AS were observed. This finding could also render a molecular explanation for the clinical observation and for our results on sex-specific cardiac mitochondrial respiration in patients with AS. Overall, this emerging concept is highly complex and needs more in-depth analyses.

### 4.2 Relation between sex differences in mitochondrial and myocardial function

Efficient energy metabolism is essential for ensuring proper cardiac function, especially in cardiac hypertrophy and HF. A direct link exists between myocardial metabolism and cardiac function. The myocardium metabolizes substrates to generate ATP, which serves as the energy currency enabling cardiac function (27). Changes in metabolic remodeling have been described to be associated with the degree of LVH (28). In this study, we observed a higher mitochondrial respiration in female patients with AS compared with their male counterparts; one could deduce that a superior myocardial functional recovery after TAVI in women may be due to more efficient energy metabolism. Within the limited patient cohort investigated, the correlation between ejection fraction (EF) or LVM and State-3 respiration did not reach significance. However, in women, there may be an association between better mitochondrial function, better myocardial function, and recovery (29).

### 4.3 Presence and role of steroid hormone receptors in the heart

Possible sex-specific differences in the heart could be mediated by E2 and ERs. The localization of ERs appears to be tissue- and cell -specific. Furthermore, Nordmeyer et al. (6) demonstrated that both receptors ERα and ERβ are expressed in human hearts and their expression is significantly increased in the hearts of patients with AS. Many subsequent studies investigated the role of ERs in the heart, revealing a variety of functions in diverse molecular signal transduction pathways and cell types (16, 30). Estrogen mediates genomic and non-genomic effects through its receptors. These effects can occur within the nucleus or at the plasma membrane, for instance, by estrogen binding to its receptors, enabling interaction with specific estrogen-responsive elements on DNA to regulate specific gene transcription (30). Besides the numerous roles that estrogen and its receptors play on the plasma membrane and within the nucleus, emerging evidence suggests that mitochondria represent another site of action for this sex hormone (31).

### 4.4 Presence and role of ERβ

Key enzymes in glucose and fatty acid metabolism are regulated by sex and estrogen (32–35). Metabolic gene deletion led to sex-specific phenotypes with HF-related death in male animals and survival in females (36). A central coactivator in the regulation of these genes, peroxisome proliferator-activated receptor gamma coactivator 1-alpha (PCG-1a), is regulated by ERβ and E2 (8, 32, 33). We and others confirmed a major role of ERβ in the modulation of metabolic genes and myocardial hypertrophy (37, 38). Deletion of ERβ in a pressure overload model had detrimental effects on the development of HF in mice. ERβ had a cardioprotective role in female mice, but not in male mice (7).

Both ERα and ERβ, in addition to being found in the nuclei and plasma membrane, have also been identified in the mitochondria of many different cell types and species (for details, see this review) (39). It seems so that mitochondrial location of E2 receptors seems to be tissue -specific. The presence of ERβ was detected in human heart mitochondrial proteins by Yang et al. (40) in 2004 while not detected in liver cells by the same methods (41). However, ERβ seems to be the main ER present in mitochondria as demonstrated by immunohistochemistry, immunocytochemistry, and immunoblots using a large panel of antibodies, and mechanisms of import have been studied [see (42) for review]. The mitochondrial localization of ERβ is the prerequisite for its functional effects. As already reported for other protective mechanisms, recovery of mitochondrial respiration takes place only in females (49–51).

The mitochondrial localization of ERβ might explain why only E2 and the ERβ agonist—and not the ERα agonist—were able to restore State-3 respiration following the GDX in this study. As previously reported for other protective mechanisms, recovery of mitochondrial respiration occurs exclusively in females (49–51). Unfortunately, it is not specified whether this research on ERβ localization in human cardiac mitochondria utilized male or female tissues.

Our understanding of sex differences is still incomplete and highlights the importance of developing mechanistic studies to delineate more precisely sex hormone receptor expression, localization, and function in male and female heart and skeletal muscles. Also, the effects of other sex hormones, such as androgens, which were not included in this study, need to be investigated.

## 5 Limitations

We explored whether the observed sex difference in mitochondrial respiration could be attributed to the action of estrogen and its receptors. Unfortunately, these studies could not be conducted on human cardiac specimens due to the severe limitations in the number and size of human biopsies. Hence, we utilized a mouse model to substantiate this possibility. Moreover, the duration of E2 and ER agonist treatment was 24 h; this short period of time was insufficient to induce a measurable estrogen effect on uterine weight.

## 6 Conclusion

Considering the pronounced sex-specific regulation of cardiac mitochondrial function, we anticipate the present findings to contribute to major underlying mechanisms responsible for the sex differences in clinical outcomes of patients with AS and those associated with progression to heart failure. We put forward that sex-specific therapeutic interventions may be of increased value toward a more personalized medical care.

The current study demonstrates that cardiac mitochondrial respiration varies between men and women under pathological conditions, such as AS, and in mice under physiological conditions. The observed sex-specific differences on mitochondrial functions may be mediated by E2 and ERβ, and not ERα.

## Data availability statement

The original contributions presented in the study are included in the article/supplementary material. Further inquiries can be directed to the corresponding author.

## Ethics statement

The studies involving humans were approved by the Charité University Hospital Ethics Committee and complies with the principles outlined in the Declaration of Helsinki. The studies were conducted in accordance with the local legislation and institutional requirements. The participants provided their written informed consent to participate in this study. The animal study was approved by the EU Directive 2010/63/EU for animal experiments and approved by the Landesamt für Gesundheit und Soziales, Berlin, Germany (G0027 - 11) and followed the ‘Principles of Laboratory Animal Care’ (NIH publication no. 86 - 23, revised 1985) as well as the current version of German Law on the Protection of Animals. The study was conducted in accordance with the local legislation and institutional requirements.

## Author contributions

DF and VR-Z contributed to the study conception and design. Material preparation, experimental performance and data collection & analysis were performed by AE and AA. GP collected patient consent for obtaining human biopsies and data. DF and AE analyzed the data. DF drafted the manuscript. AA, AE, and VR-Z revised critically the manuscript. All authors contributed to the article and approved the submitted version.
